# Comparison of dynamic contrast-enhanced MR, ultrasound and optical imaging modalities to evaluate the antiangiogenic effect of PF-03084014 and sunitinib

**DOI:** 10.1002/cam4.215

**Published:** 2014-02-27

**Authors:** Cathy C Zhang, Zhengming Yan, Anand Giddabasappa, Patrick B Lappin, Cory L Painter, Qin Zhang, Gang Li, James Goodman, Brett Simmons, Bernadette Pascual, Joseph Lee, Ted Levkoff, Tim Nichols, Zhiyong Xie

**Affiliations:** 1 Oncology Research Unit, Pfizer Global Research and DevelopmentLa Jolla, California; 2 Comparative Medicine, Pfizer Global Research and DevelopmentLa Jolla, California; 3 Drug Safety, Pfizer Global Research and DevelopmentLa Jolla, California; 4 Precision Medicine, Pfizer Global Research and DevelopmentGroton, Connecticut

**Keywords:** Antiangiogenesis, imaging, PF-03084014, sunitinib, *γ*-secretase inhibitor

## Abstract

Noninvasive imaging has been widely applied for monitoring antiangiogenesis therapy in cancer drug discovery. In this report, we used different imaging modalities including high-frequency ultrasound (HFUS), dynamic contrast enhanced-MR (DCE-MR), and fluorescence molecular tomography (FMT) imaging systems to monitor the changes in the tumor vascular properties after treatment with *γ*-secretase inhibitor PF-03084014. Sunitinib was tested in parallel for comparison. In the MDA-MB-231Luc model, we demonstrated that antiangiogenesis was one of the contributing mechanisms for the therapeutic effect of PF-03084014. By immunohistochemistry and FITC-lectin perfusion assays, we showed that the vascular defects upon treatment with PF-03084014 were associated with Notch pathway modulation, evidenced by a decrease in the HES1 protein and by the changes in VEGFR2 and HIF1*α* levels, which indicates down-stream effects. Using a 3D power Doppler scanning method, ultrasound imaging showed that the% vascularity in the MDA-MB-231Luc tumor decreased significantly at 4 and 7 days after the treatment with PF-03084014. A decrease in the tumor vessel function was also observed through contrast-enhanced ultrasound imaging with microbubble injection. These findings were consistent with the PF-03084014-induced functional vessel changes measured by suppressing the *K*^trans^ values using DCE-MRI. In contrast, the FMT imaging with the AngioSence 680EX failed to detect any treatment-associated tumor vascular changes. Sunitinib demonstrated an outcome similar to PF-03084014 in the tested imaging modalities. In summary, ultrasound and DCE-MR imaging successfully provided longitudinal measurement of the phenotypic and functional changes in tumor vasculature after treatment with PF-03084014 and sunitinib.

## Introduction

The Notch pathway plays an important role in tumor angiogenesis 1. Elevated expression of Notch ligand Jagged1 was reported in various malignancies. In vivo deletion of Jagged1 gene resulted in dramatically reduced endothelial sprouting 2. Another Notch ligand, Dll4, is also a critical vascular function mediator. When treated with a Dll4 therapeutic antibody, endothelial cells become hyperproliferative leading to a capillary and defective vascular phenotype 3. Notch regulates the interactions between endothelial and tumor cells, which enables tumor cells to escape from dormancy 4. During tumorigenesis, Notch crosstalks with VEGF signaling and mediates tumor vessel sprouting 5,6. Abundant evidence has shown that treatment with Notch inhibitors results in tumor vascular defect 7–9.

In addition to its critical involvement in angiogenesis, Notch pathway also mediates tumorigenesis and progression through tumor cell proliferation, apoptosis, and self-renewal 10,11. Notch pathway activation is a multistep process in which Notch ligands, Notch receptors as well as proteases all play critical roles. As a result, diverse approaches have been actively pursued to inhibit Notch pathway activation in both endothelial cells and tumor cells to achieve anticancer therapy. Upon activation of Notch pathway, *γ*-secretase cleaves Notch receptor and subsequently releases the notch intracellular domain, which enters the cell nucleus to trigger oncogenic activation. PF-03084014 is a *γ*-secretase inhibitor that inhibits Notch pathway and exhibits antitumor efficacy in a panel of breast cancer models 12. In combination with docetaxel, PF-03084014 significantly enhanced chemotherapy against breast cancer xenograft growth through diverse mechanisms including antiproliferation, apoptosis induction and inhibition of tumor cell self-renewal 13. In this report, we aimed to evaluate the antiangiogenesis effect of PF-03084014 using a variety of *ex vivo* and in vivo live imaging analyses.

Since the FDA approval of bevacizumab, antiangiogenesis has become a widely accepted strategy for anticancer therapy 14. However, in recent years, clinical experience of antiangiogenic therapy has exposed some limitations such as post therapy related tumor invasiveness, equivocal long-term benefit and drug resistance. Future applications of antiangiogenic agents would require rational combination strategy to circumvent or alleviate issues associated with monotherapy. To achieve this, it is desirable to develop robust biomarkers for these agents to increase confidence in the mechanism and provide guidance for dosing optimization. One of the commonly used approaches to monitoring antiangiogenic effect is IHC staining of CD31 on tumor biopsy 15. However, success with the use of CD31 staining to measure the therapy associated with micro-vascular density (MVD) change has been very limited 16. In addition, multiple samplings are unethical and impractical. On the other hand, the application of live imaging allows longitudinal measurement of the treatment effect on tumor vascular properties noninvasively. While optical imaging is primarily restricted to rodent models 17, other imaging modalities such as high-frequency ultrasound (HFUS), dynamic contrast enhanced-magnetic resonance imaging (DCE-MRI) and computed tomography (CT) imaging have been frequently utilized in both preclinical and clinical settings 18–21. Two studies provide examples of functional imaging application in the clinical setting 22,23. The use of dynamic contrast-enhanced ultrasonography (DCE-US) successfully predicted the efficacy of sunitinib. The imaging modalities including functional ultrasound, DCE-MR, and CT imaging empowered by contrast agents permits the measurements of tumor perfusion or permeability. These imaging technologies provide an early measurement of vascular functional changes after antiangiogenic therapy before the tumor size change becomes apparent.

In this report, we evaluated the antiangiogenic phenotypes of PF-03084014 in the MDA-MB-231Luc model. Aside from the tumor histological analysis, HFUS, DCE-MR, and fluorescence molecular tomography (FMT) imaging systems were utilized to noninvasively assess the treatment effect of PF-03084014. Sunitinib was used as a comparator for different imaging endpoints.

## Materials and Methods

PF-03084014 and sunitinib were synthesized by Pfizer chemists. Unless otherwise noted, all chemicals were purchased from Sigma-Aldrich (St. Louis, MO). MDA-MB-231Luc and AngioSense 680 EX was purchased from PerkinElmer (Waltham, MA). The antibodies for IHC analyses were anti-BrdU (BD Pharmingen, San Diego, CA), anti-HIF1*α* (R&D Systems, Minneapolis, MN), anti-phospho-H2AX, anti-HES1, and anti-VEGFR2 (Cell Signaling Technology, Danvers, MA).

### In vivo studies and drug administration

All animal experimental procedures complied with the Guide for the Care and Use of Laboratory Animals (Institute for Laboratory Animal Research, 1996) and were approved by the Pfizer Global Research and Development Institutional Animal Care and Use Committee. Two million MDA-MB-231Luc cells were subcutaneously implanted in the dorsal region of female SCID-beige mice (Charles River, San Diego, CA). Mice with palpable tumors were randomly assigned into different groups such that the mean value of tumor size was same between groups. Mice were then p.o. administered with: (1) vehicle; (2) PF-03084014 at 110 mg/kg twice daily; and (3) sunitinib at 60 mg/kg once daily for up to 12 days. Pharmacodynamic analysis or imaging scan was performed at specified time points during the treatment period. Tumors were measured two to three times weekly using calipers and tumor volume was calculated as 0.5 × [length × width^2^].

### Immunohistochemical staining

Tumor samples were collected and prepared into formalin-fixed, paraffin-embedded tissue blocks. The staining procedure was performed according to the manufacturer's instructions. The frequency of positive cells was scored semiquantitatively by board-certified pathologists. Identification of tumor necrosis was performed using eCognition image analysis technology (Definiens, Munich, Germany).

### Lectin perfusion assay

For functional tumor vasculature assessment, tumor-bearing mice received an i.v. injection of 5 mg/kg FITC-lectin (Vector Labs, Burlingame, CA) 10 min prior to euthanasia. The tumor samples were frozen in OCT® medium, cryosectioned into 100 *μ*m slices, and subsequently stained with anti-CD31 antibody. The fluorescence images were captured using a Nikon Eclipse TE2000 fluorescent microscope with Q-Capture software, and the analysis of microvessel density was performed using Image Pro Plus 5.1 (Media Cybernetics, Silver Spring, MD).

### High-frequency ultrasound imaging

Ultrasound imaging was performed using a Vevo™ 770 system (VisualSonics, Inc., Toronto, ON, Canada) with a 40 MHz high frequency transducer (RMV 706). Mice anesthetized with 2% isoflurane were placed on a homoeothermic imaging stage. Coupling gel was applied on the top of tumor to create a 3–5 mm acoustic standoff between the transducer and the tumor. For contrast imaging, the system was set on contrast mode and scanned manually through the tumor to establish a central field of view. The images were acquired before and after a bolus injection (intravenous) of 100 *μ*L non-targeted microbubble contrast agent (VisualSonics, Inc.). To perform 3D-Power Doppler imaging, each tumor was prescanned in B-mode to define adequately the boundary of the tumor mass based on echogenicity parameters. Serial 2D images with the scanning frequency of every 0.5 mm were acquired throughout the entire tumor for 3D reconstruction. The 3D volume was analyzed by drawing a region of interest (ROI) without interferences by large echoes. The 3D imaging provides the total tumor vessel volume normalized by the tumor volume (% vascularity). All the HFUS data analyses were performed using Vevo 770 3.0.0 software.

### Fluorescence molecular tomography imaging

FMT 2500 (PerkinElmer) was used to quantify the tumor vessel density and angiogenesis. The tumor uptake and kinetics of fluorescent vascular probe AngioSense 680 EX (PerkinElmer) were assessed. A pilot study determined that the best time point for quantitative measurement of angiogenesis and blood vessel density was 24 h post–tail vein injection. Briefly, the mice were anesthetized using 2% isoflurane and carefully positioned in the imaging cassette, which was then placed into the FMT imaging chamber. The images were acquired at medium resolution and the entire image acquisition sequence took approximately 4–5 min per mouse. The collected fluorescence data were reconstructed by TrueQuant 3.0 software for the quantification and comparison of three-dimensional fluorescence signal within the tumors. Standardized fluorescence values (in picomoles/mm3) were calculated by dividing the total tumor fluorescence intensity by the tumor volume assessed by caliper.

### DCE-MRI study

DCE-MRI was conducted on a BioSpec 7T MRI system (Bruker BioSpin, Billerica, MA). Images were acquired with 25 mm quadrature transmit-receive volume RF coil (Doty Scientific, Columbia, SC). Coronal spin echo and transversal multislice fast spin echo T2-weighted images were acquired for tumor localization and delineation. A variable repetition-time (TR) saturation-recovery sequence was used to measure the pre-contrast T1 value. For DCE-MRI, a series of multislices T1-weighted spoiled gradient echo images (Bruker-Paravision FLASH sequence; repetition-time, 108.7 msec; echo-time, 2.2 msec; flip-angle, 30 degrees; matrix size, 64 × 128; spatial resolution, 0.4 × 0.2 mm^2^; six slices with distance of 1.5 mm; temporal resolution, 5 sec/image) were acquired over 20 min. Gd-DTPA (Magnevist, Berlex Inc., Montville, NJ) was injected 4 min after initiation of the image series. Tumor was manually delineated from the T2-weighted images and volume of the tumor was computed based on the segmentation. *K*^trans^, a transfer constant of contrast between the blood stream and the extracellular space, was voxel-wisely computed from the time course of the image intensity change in DCE-MR images 24.

### Statistical analysis

Statistical analyses were conducted using GraphPad Prism 5 (Graph pad Prism, San Diego, CA). The tumor sizes of vehicle and treated groups were compared using Student's t-test (two-sided *P*-value).

## Results

### Characterization of the tumor vasculature in the MDA-MB-231Luc tumor model

**Figure 1:**
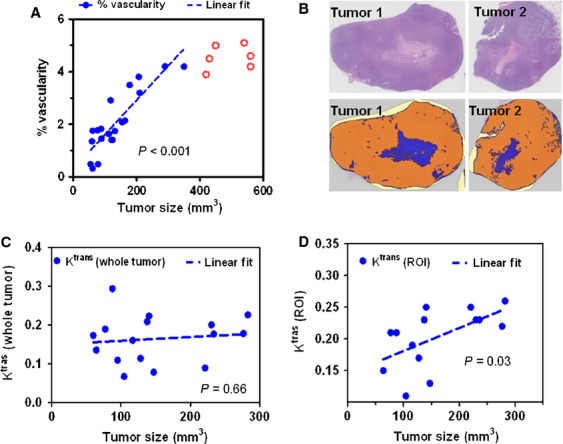
Characterization of nontreated MDA-MB-231 Luc tumor vasculature. (A) Power Doppler ultrasound imaging was performed to assess the tumor vascularity. (B) H & E staining (top panel) by Definiens imaging (bottom panel) analysis depicts the increased necrosis region (blue section in bottom panel) when tumor size was enlarged. DCE-MRI was performed to measure the *K*^trans^ values. The tumor sizes failed to show a correlation with the *K*^trans^ values from the whole tumor (C), whereas demonstrated a linear correlation with *K*^trans^ values from ROIs (D).

To avoid assay variability during therapy assessment, nontreated tumor bearing mice were evaluated to understand the relation between tumor size and the vascular property. Power Doppler ultrasound imaging was performed to assess the tumor vascularity by using a 3D scanning method. For tumors smaller than 400 mm^3^, a positive correlation (*P* < 0.01) was observed between the tumor size and the percent vascularity (Fig. 1A). In tumors larger than 400 mm^3^, the %vascularity no longer increased proportionally, possibly due to increased necrosis. To test this hypothesis, tumors in the range of 100–400 mm^3^ were collected for H& E staining. Based on Definiens imaging analysis, increased necrosis (blue section) was observed when the tumors became enlarged (Fig. 1B). These data suggest that smaller tumors (<400 mm^3^) are preferred for evaluating therapeutic associated changes in tumor vasculature.

DCE-MRI was performed to measure the *K*^trans^ values, which provide a physiological measure of the tumor vascular function through measuring the tumor uptake and clearance of contrast agents. In these nontreated mice, the K^trans^ value from the whole tumor failed to show a correlation with the tumor size (Fig. 1C). In contrast, the *K*^trans^ values from the superficial region (ROI) demonstrated a positive correlation (*P* = 0.03) with the tumor sizes (Fig. 1D), indicating a higher perfusion rate on the tumor surface. Therefore, we utilized *K*^trans^ value from the surface ROI as a surrogate measure of antiangiogenic effect after therapy.

### The antitumor efficacy of PF-03084014 and the associated mechanism

**Figure 2:**
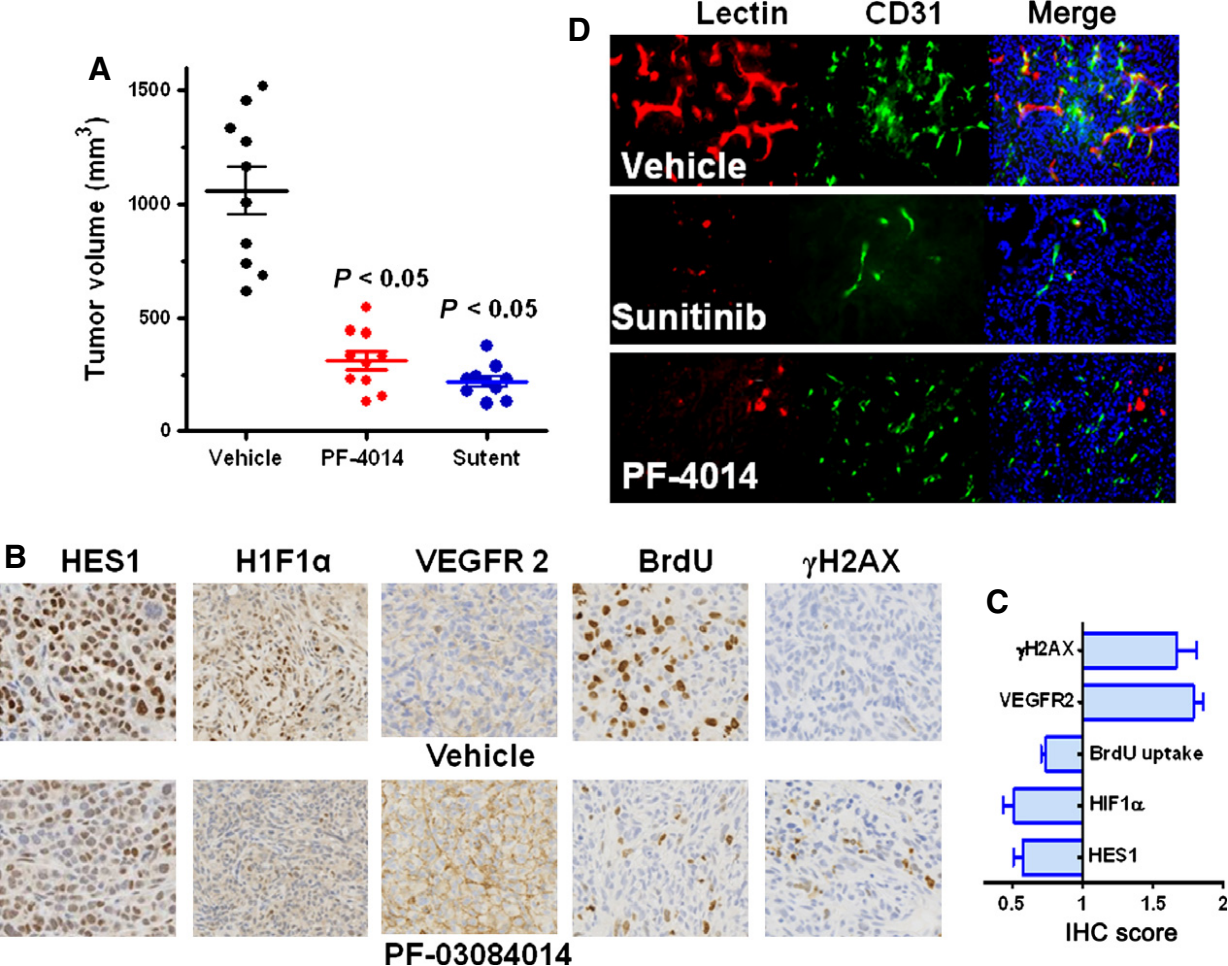
Antitumor efficacy and pharmacodynamic assessment of PF-03084014 in the MDA-MB-231Luc xenograft model. Mice bearing palpable tumors were p.o. administered with P-F03084014 (110 mg/kg) twice daily or sunitinib (60 mg/kg) once daily until ready for analysis. (A) PF-03084014 and sunitinib exhibit significant (*P* < 0.05) antitumor efficacy. This graph indicates the caliper measurement after a continuous 12-day treatment period. *N* = 10 mice/group; (B) Representative IHC images of the treatment induced changes in HES1, HIF1*α*, VEGFR2, BrdU, and *γ*H2AX levels. (C) The semiquantitative analysis of the IHC scores for the tested markers was performed by a board-certified pathologist. The graph represents the value relative to vehicle treatment (=1). Value = mean ± SEM. The results indicated that all changes between the vehicle and PF-03084014 treatment were significant (*P* < 0.05). (D) PF-03084014 or sunitinib impairs functional vasculature as measured by the lectin-perfusion assay. After tumor-bearing mice were treated daily for 4 days, mice were i.v. injected with 5 mg/kg FITC-lectin prior to tumor collection. In C and D, *N* = 5 mice/group.

The antitumor efficacies of sunitinib and PF-03084014 were assessed in the MDA-MB-231Luc model. Mice with palpable tumors were randomly assigned into three groups and treated continuously for 12 days. Both PF-03084014 and sunitinib demonstrated significant (*P* < 0.05) antitumor efficacies (Fig. 2A). Pharmacodynamic study of PF-03084014 was performed in a separate cohort of mice. Tumors were harvested on day 4 post dosing initiation and subject to IHC analysis (Fig. 2B). PF-03084014 suppressed the Notch down-stream protein HES1, suggesting target associated antitumor efficacy. Concurrently, a reduction of HIF1*α* and BrdU uptake, as well as an increase in the VEGFR2 and *γ*H2AX levels was observed after treatment. Semiquantitative analysis of the IHC scores for the tested markers (Fig. 2C) suggested that all treatment associated changes were statistically significant (*P* < 0.05).

### Functional vasculature changes by lectin perfusion assay

We performed lectin perfusion assay to assess whether the efficacy of PF-03084014 or sunitinib was associated with the microvessel density change in vivo. Tumor bearing mice were assigned into three groups and treated continuously with vehicle, sunitinib and PF-03084014. On day 4 a fluorescently conjugated FITC-lectin was i.v. injected and tumors were collected at 10 min post injection. Tumors were then sectioned for immunofluorescence costaining of FITC-lectin and CD31. PF-03084014 minimally impacted the microvessel density of CD31 staining, whereas it diminished functional lumens stained with FITC-lectin (Fig. 2D and Fig. S1), which is consistent with the observations in other models 12. In contrast, sunitinib at effective doses clearly showed a distinct phenotype. Both FITC-lectin and CD31 positive staining was markedly reduced after treatment.

### Fluorescence tomography imaging in MDA-MB-231Luc tumor-bearing mice

**Figure 3:**
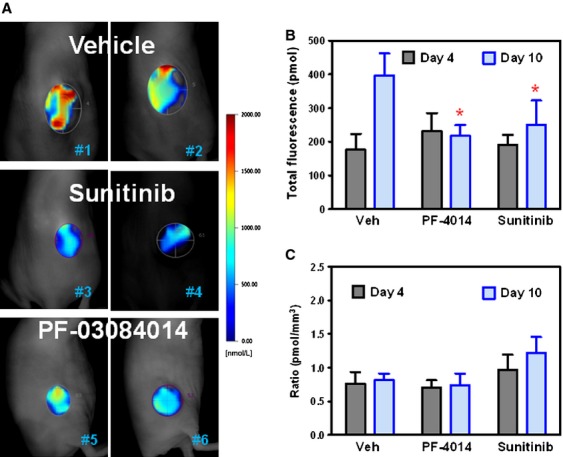
FMT quantitative imaging analysis of the changes in vascular density after treatment with PF-03084014 and sunitinib in the MDA-MB-231 Luc xenograft model. Tumor bearing mice under treatment were imaged on day 4 and day 10 after dosing initiation. At 24 h before the imaging scan, mice were i.v. injected with AngioSense680 EX. *N* = 8 mice/group. In B and C, values are expressed as the mean ± SEM. (A) Representative FMT images from each group on day 10. (B) The total florescence was quantified by the total pixels within the regions of interest around each tumor. (C) The relative tumor vascular density was estimated by the total fluorescence values normalized to tumor volume (pmoles/mm^3^).

The effect of sunitinib and PF-03084014 on tumor vascular density was also assessed by FMT imaging. On day 4 and day 10 post dosing initiation, mice were iv injected with AngioSense680 EX. FMT imaging was performed 24 h later. Figure 3A depicts the representative images of tumor-bearing mice in each group on day 10. The total tumor fluorescence showed no difference between the treated and untreated groups on day 4 (Fig. 3B). In contrast, the reduction in total fluorescence by either PF-03084014 or sunitinib treatment was significant (*P* < 0.05) on day 10 when the tumor volumes in treated mice were also statistically smaller compared to vehicle group. However, the tumor vascular density, which was the total fluorescence values normalized to tumor volume (pmoles/mm^3^), exhibited no significant change in either group that received treatment compared to the vehicle group (Fig. 3C).

### HFUS imaging demonstrates the suppression of tumor vascular volume as well as vascular function after therapy

**Figure 4:**
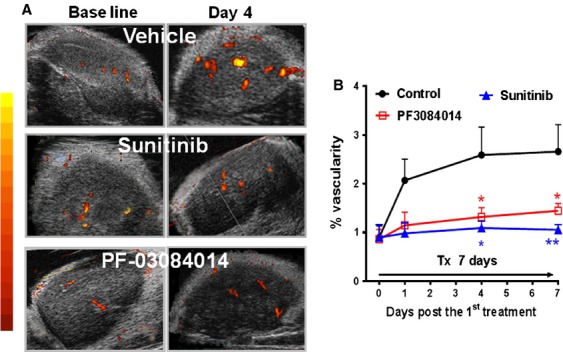
Serial power Doppler ultrasound imaging of the MDA-MB-231 Luc tumor during PF-03084014 or sunitinib therapy. Tumor bearing mice under treatment were imaged on days 1, 4, and 7 after initiating treatment. *N* = 6 mice/group. (A) Representative images of each group on day 0 (baseline) and day 4 via power Doppler analysis. (B) The time-dependent changes of the%vascularity in each group. *N* = 6 mice/group. Values are presented as the mean ± SEM.

To further investigate the phenotype change in tumor vasculature after treatment with PF-03084014 and sunitinib, power Doppler ultrasound imaging was performed. Mice bearing palpable MDA-MB-231Luc tumor sizes were randomized and treated with vehicle, PF-03084014 and sunitinib. Serial images were acquired at baseline and during treatment. A sign of decreased tumor vascular volume became noticeable on day 1 (24 h) after PF-03084014 treatment. Figure 4A depicts the representative images from each group on day 0 and day 4. Based on the quantitative analysis from the 3D images, PF-03084014 caused a significant (*P* < 0.05) suppression of the tumor vascularity on day 4 and day 7 compared to vehicle treatment (Fig. 4B). Sunitinib demonstrated a similar effect.

**Figure 5:**
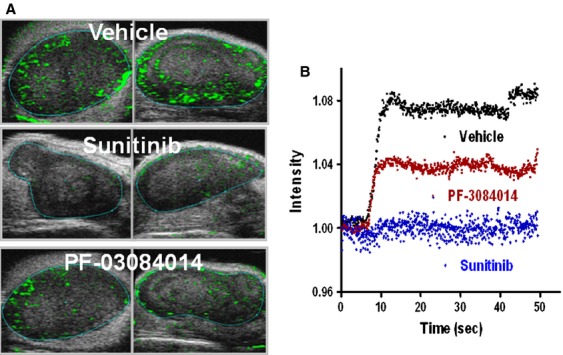
Contrast-enhanced ultrasound imaging to detect the functional vasculature changes in PF-03084014 or sunitinib treated MDA-MB-231Luc tumor. Mice bearing tumors in the range between 250–400 mm^3^ treated until day 4. Mice were i.v. injected with 100 *μ*L micro-bubble solution prior to imaging. *N* = 6 mice/group. (A) Representative images of each group. (B). Time-intensity curves depict the blood vessel perfusion rate change after the microbubble injection.

We also performed contrast-enhanced ultrasound (CEUS) imaging with microbubble contrast agent to assess the functional change in the tumor vasculature. On day 4 post initial dosing, the tumor-bearing mice were i.v. injected with 100 *μ*L nontargeted microbubble solution. Immediately following injection, the blood perfusion rate and the kinetics were acquired on a preselected 2D view for each individual tumor. Representative images were captured at the peak blood flow rate from each group of mice (Fig. 5A). A quantitative measure of the time-dependent microbubble perfusion rate reflected the treatment-induced changes in the tumor perfusion kinetics (Fig. 5B). Both treated groups demonstrated significant lower values compared to vehicle treatment (*P* < 0.05). Clearly the CEUS imaging demonstrated the antiangiogenic effects of both PF-03084014 and sunitinib. Compared to PF-03084014, sunitinib caused a higher magnitude change of the functional vasculature.

### *K*^trans^ value changes via DCE-MRI reflect the treatment associated changes in tumor vessel function

**Figure 6:**
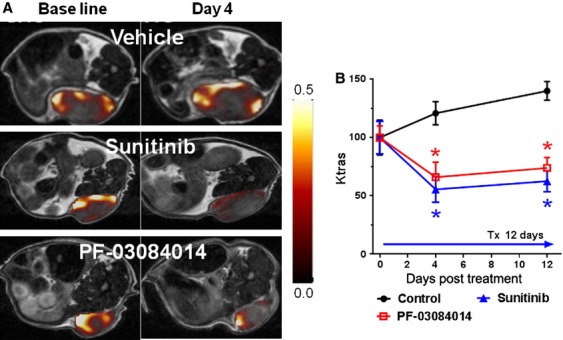
The effects of PF-03084014 and sunitinib on MDA-MB-231Luc tumor vasculature using DCE-MRI. Tumor bearing mice under treatment were imaged on day 0, 4, and 10 after dosing initiation. (A) Representative images of *K*^trans^ maps of each group on days 0 and 5. (B) The time course of the changes in the *K*^trans^ (ROI) values demonstrate the treatment effect on functional tumor vasculatures. *N* = 6 mice/group. Values are presented as the mean ± SEM.

DCE-MRI was performed to obtain the treatment associated *K*^trans^ value changes as a physiological measure of the tumor vascular function. The MDA-MB-231Luc tumor bearing mice were scanned before dosing and on day 4 and day 12 post-treatment with PF-03084014 or sunitinib. Figure 6A depicts the representative images of the *K*^trans^ map from vehicle and treated mice at baseline and day 4 after dosing commencement. To avoid potential data variability from the tumor necrotic core, ROIs around the rim of the tumor were selected for computing the mean *K*^trans^ value.

The percentage changes in *K*^trans^ value (ROI) relative to the baseline measurement are displayed in Figure 6B. DCE-MRI detected significantly (*P* < 0.05) lower *K*^trans^ values in tumors treated with PF-03084014 (31.2% decrease) or sunitinib (47.3% decrease), compared to baseline measurement, whereas a 20.8% increase of *K*^trans^ in the vehicle group was observed. On day 12, *K*^trans^ values in the PF-03084014 and sunitinib treated mice remained at similar levels. By comparison, the vehicle treated mice exhibited a significant higher *K*^trans^ value compared to baseline measurements. Overall, the *K*^trans^ value in both treated groups was significantly (*P* < 0.05) less than that of the vehicle group on day 4 and day 12 after treatment, suggesting an antiangiogenic response to PF-03084014 or sunitinib.

## Discussion

In this study, multiple imaging modalities were used to assess the phenotypic and functional changes of tumor vasculature after PF-03084014 or sunitinib treatment. Pharmacodynamic assessments provided the correlation between Notch target modulations and the changes in tumor vascular characteristics. We explored the strengths and weaknesses of various imaging modalities and evaluated how to apply these tools to best assess angiogenesis therapy.

In light of the critical role that Notch plays during angiogenesis, impairing tumor vasculature was shown to be a phenotype when PF-03084014 inhibits Notch signaling in breast cancer xenografts 12. In addition, PF-03084014 also elicits other activities including antiproliferation and apoptosis, as previously reported 12,13. These pharmacodynamic changes were accompanied by decreased HES1 protein level, suggesting the Notch target correlation with the phenotype change because HES1 is a Notch target protein. The functional vasculature changes by PF-03084014 simultaneously occurred with increased VEGFR2 and decreased HIF1*α* levels, indicating an opposing effect between the Notch and VEGF inhibitors in the regulation of angiogenesis. As HIF1*α* and its target genes can be activated by Notch signaling 25, the modulation of HIF1*α* was expected upon treatment with PF-03084014. In contrast, bevacizumab, a VEGF antagonist, induced upregulation of HIF-1 26,27. According to existing reports, *γ*-secretase inhibitors stimulate an elevation of the tumor VEGFR2 level 28,29 through a feedback mechanism 30 when Notch crosstalks with VEGF signaling. These results are consistent with our observations with PF-03084014.

The antiangiogenic effect of PF-03084014 was previously reported 12. Unlike the treatment effect by sunitinib, the vasculature phenotype change by PF-03084014 was not accompanied by a corresponding decrease in CD31 staining, presumably due to the opposing effect of Dll4 3 and Jagged1 on angiogenesis 1,7. Because blocking the Notch pathway by PF-03084014 resulted in a capillary vasculature phenotype and decreased tumor vessel growth 12, tumor vasculature was defective despite unchanged CD31 positivity. Thus, CD31 staining did not correlate with any Notch-mediated MVD changes after PF-03084014 therapy. By using an *ex vivo* lectin perfusion assay, we observed a decrease in micro-vessel density after therapy. The caveat for the 2D *ex vivo* MVD assessment was the quantitation process. A series of histological slices were extensively evaluated to perform a semiquantitative analysis, particularly for PF-03084014, because the treatment effect on MVD was less drastic compared to sunitinib.

The results from the in vivo HFUS and DCE-MRI imaging analyses were consistent with the outcome of lectin perfusion assay. More importantly, these imaging modalities provided dynamic and quantitative measures of the antiangiogenic effect of PF-03084014 and sunitinib. Ultrasound imaging offered both endpoints including % vascularity and vascular perfusion rate to detect the morphologic and functional changes in tumor vasculature after therapy. The therapy-associated functional changes in tumor vasculature were further confirmed by the assessment of *K*^trans^ values by DCE-MRI imaging. Both US and DCE-MRI modalities showed advantages for offering a longitudinal and quantitative assessment of the changes in tumor vasculature after treatment, and they provided high confidence in these mechanisms.

Given the fact that tumor size increase in general leads to an increase in necrotic region, it is important to avoid any necrosis-associated variability during imaging acquisition. In the process of CEUS imaging, the tumor vascular perfusion kinetics exhibited high variability when MDA-MB-231Luc tumor grew beyond 400 mm^3^ (data not shown), presumably due to increased necrotic fraction, as observed in the *ex vivo* H&E staining. This notion was further supported by the 3D ultrasound assessment that the vascular fraction, or the % tumor vascularity of the whole tumor, no longer increased based on tumor size after exceeding 400 mm^3^. Although the transducer (40 MHz) showed a relatively high spatial resolution during US imaging, DCE-MRI offered better soft tissue contrast; therefore, we easily identified the tumor boundary, as reported in another subcutaneously implanted tumor model 31. Therefore, to evaluate therapy, DCE-MRI showed the advantage for allowing *K*^trans^ analysis with accurate selection of ROIs, often in the peripheral region where tumors are highly vascularized 32–34. Consistent with these reports, the *K*^trans^ value in the rim of MDA-MB-231Luc tumor increased accordingly when tumor sizes were enlarged during the 12-day observation period, whereas this correlation was not observed using the whole tumor.

Despite the vascular alteration detected by multiple assays, FMT imaging failed to monitor such changes after treatment with PF-03084014 or sunitinib. However, in a previous report on FMT imaging application 35, it was noted that the AngioSense750 probe retained inside the tumor vessel at 4 h postinjection leaked out after 24 h. According to Zhang et al. 35, the functional vasculature changes post anti-VEGF therapy were predicted by the FMT imaging at 4 h post probe injection. In our in vivo imaging study, we were only able to quantify the tumor specific retention of AngioSense at 24 h, as early scan (6 h or less) led to strong signal interference by the nonspecific distribution of the fluorescence probe in normal organs. As the therapy-associated changes on tumor vasculature by PF-03084014 or sunitinib were successfully measured by other imaging modalities, the FMT imaging readout of AngioSense in the MDA-MB-231Luc was unlikely to be vascular specific, or it is likely that the tracer was largely infiltrated in the interstitial space rather than staying inside the tumor vasculature.

In summary, we have demonstrated the antiangiogenic effect of PF-03084014 through *ex vivo* analyses and in vivo imaging technology. Sunitinib was used as a reference to validate the treatment related phenotype changes in the anatomic and functional vasculature. These results provide insight into the application of in vivo imaging technology during the drug discovery process.

## Supporting Information

**Figure S1.** The semiquantitative analysis of the microvessel density using CD31 and FITC-lectin staining. The graph represents the number of microvessels per field. Value = mean ± SEM. *N* = 5 mice/group. *: *P* < 0.05 versus vehicle treatment.
