# Supplementary figures and images for: Comparison of dynamic contrast-enhanced MR, ultrasound and optical imaging modalities to evaluate the antiangiogenic effect of PF-03084014 and sunitinib
Source: Cancer Med. 2014 Feb 27;3(3):462–71. doi: 10.1002/cam4.215 (PMC4101737; doi:10.1002/cam4.215)

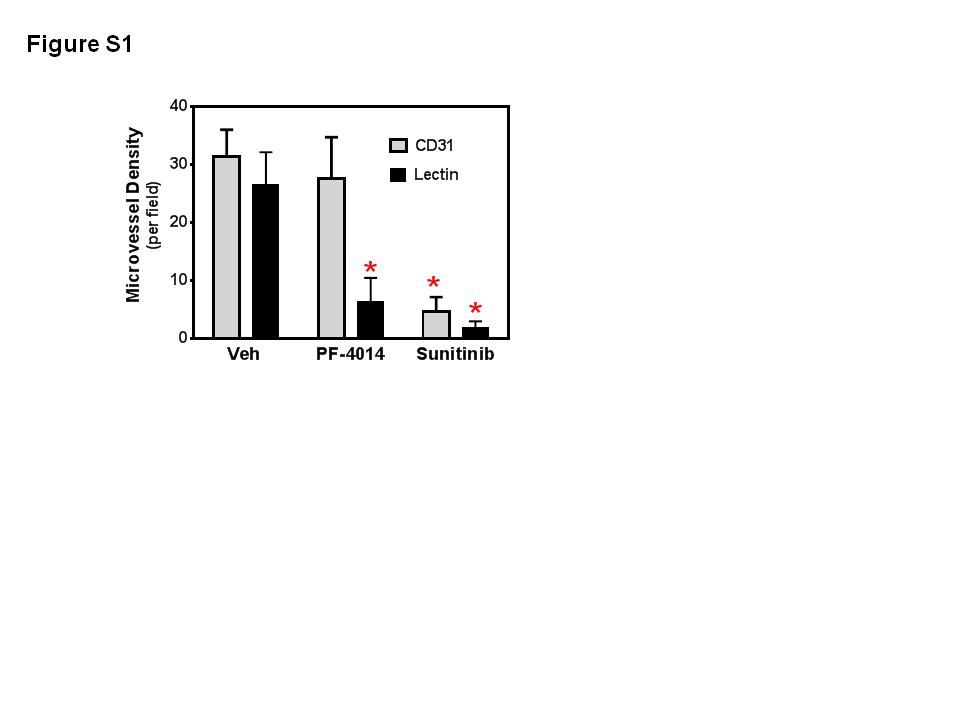

Supplement: Supplementary file 1 — Figure S1. The semiquantitative analysis of the microvessel density using CD31 and FITC-lectin staining. The graph represents the number of microvessels per field. Value = mean ± SEM. N = 5 mice/group. *: P < 0.05 versus vehicle treatment. [file cam40003-0462-SD1.tif]
